# Deep learning-derived cardiovascular age shares a genetic basis with other cardiac phenotypes

**DOI:** 10.1038/s41598-022-27254-z

**Published:** 2022-12-31

**Authors:** Julian Libiseller-Egger, Jody E. Phelan, Zachi I. Attia, Ernest Diez Benavente, Susana Campino, Paul A. Friedman, Francisco Lopez-Jimenez, David A. Leon, Taane G. Clark

**Affiliations:** 1 Faculty of Infectious and Tropical Diseases, London School of Hygiene & Tropical Medicine, London, UK; 2 Department of Cardiovascular Medicine, Mayo Clinic College of Medicine, Rochester, MN USA; 3 Laboratory of Experimental Cardiology, University Medical Center Utrecht, Utrecht, Netherlands; 4 Faculty of Epidemiology and Population Health, London School of Hygiene & Tropical Medicine, London, UK; 5 Department of Community Medicine, UiT the Arctic University of Norway, Tromsø, Norway

**Keywords:** Genome-wide association studies, Cardiovascular genetics, Machine learning, Predictive markers

## Abstract

Artificial intelligence (AI)-based approaches can now use electrocardiograms (ECGs) to provide expert-level performance in detecting heart abnormalities and diagnosing disease. Additionally, patient age predicted from ECGs by AI models has shown great potential as a biomarker for cardiovascular age, where recent work has found its deviation from chronological age (“delta age”) to be associated with mortality and co-morbidities. However, despite being crucial for understanding underlying individual risk, the genetic underpinning of delta age is unknown. In this work we performed a genome-wide association study using UK Biobank data (n=34,432) and identified eight loci associated with delta age ($p\le 5 \times 10^{-8}$), including genes linked to cardiovascular disease (CVD) (e.g. *SCN5A*) and (heart) muscle development (e.g. *TTN*). Our results indicate that the genetic basis of cardiovascular ageing is predominantly determined by genes directly involved with the cardiovascular system rather than those connected to more general mechanisms of ageing. Our insights inform the epidemiology of CVD, with implications for preventative and precision medicine.

## Introduction

For decades it has been known that a person’s electrocardiogram (ECG) changes with age^1,2^. Therefore, in light of its non-invasiveness, ease of obtainment, and consequential ubiquity, there is great potential in using the 12-lead ECG as a biomarker for physiological changes caused by ageing^3^. As these changes occur gradually and at a rate that is different between individuals, there is substantial variation in the risk of chronic disease and mortality in older populations. In order to understand the sources of this variation, several indicators for “biological age” have been investigated, including changes in telomere length^4^, the epigenome^5^, blood-derived biomarkers^6^, and the transcriptome^7^. Crucially, these markers have been shown to be only weakly correlated with each other^8^, suggesting that they do not describe the same underlying physiological processes but rather different aspects of ageing^9^. Since cardiovascular disease (CVD) is a major source of mortality and morbidity, with drastically increasing prevalence in older age^10^, the deep learning-enabled ECG-derived surrogate for cardiovascular age introduced by Attia et al.^11^ represents a valuable addition to other “ageing” metrics, with both preventative and personalised medicine benefits. Here we report the results of a genome-wide association study (GWAS) using the difference between a person’s actual age and this metric as phenotype.

Initial studies trying to link chronological age to the ECG signal mostly focused on human-defined ECG features, such as the QRS duration or the length of the PR interval^12^. However, the extraction of these features is not devoid of error^13^ and captures only a fraction of the available information. Recent developments in deep learning allowed researchers to address this limitation by adapting modern convolutional artificial neural network architectures to predict patients’ ages from their ECGs^11,14,15^. These models can be trained “end-to-end” on the raw ECG traces from which they learn to extract (and combine in a non-linear manner) the features most suitable for a prediction task. Thus, the impact of human bias is minimised and predictive power improved as all the information in the signal is taken into account. In fact, several studies have shown that deep learning models trained on ECG traces already match and in some cases even exceed the performance of medical professionals in diagnosing certain cardiac conditions^16–18^. Given the increasing prevalence of ECG data, machine learning models of such capabilities could transform predictive medicine and cardiovascular research.

In order to use ECGs for age prediction, the neural network needs to learn how the “average” ECG for a particular age group looks. Thus, when it predicts an age considerable larger than the corresponding person’s chronological age (a large “delta age ”), this might be indicative of accelerated ageing of the cardiovascular system – with implications for this individual’s health. Indeed, large delta age has been shown to be associated with CVD, treatment outcomes, and mortality^3,11,14^. This observation suggests at least two principal areas of applications for the ECG-derived age (or delta age). On one hand, it could be used in the clinic as a readily obtainable prognostic tool for screening large numbers of patients. In this capacity, delta age is conceptually similar to the “excess heart age”^19^, the discrepancy between a person’s chronological age and their “heart age” (the age corresponding to their risk of a CVD event), which has been devised as an easily interpretable measure for CVD risk^20^. However, while the excess heart age represents the increased CVD risk due to risk factors and lifestyle choices, the delta age reflects the actual functional state of the heart. Hence, in addition to clinical use cases, ECG-derived age could also complement biomarkers used in research (e.g. telomere length or the epigenetic clock, among others) for tracking ageing in general and vascular ageing in particular. One crucial advantage of the ECG-derived age over many other ageing-related biomarkers used in research is the wide-spread use of the ECG and how comparatively easy it is to obtain. This makes it especially interesting for association studies which suffer from low effective sample sizes for many disease-related phenotypes as these are usually relatively rare or can remain undiagnosed, diluting the strength of the statistical signal. Furthermore, delta age is not tied to a single type of CVD, but instead combines effects on the ECG of multiple conditions in addition to “normal” changes expected due to ageing. It might therefore lead to the discovery of genetic variants that are not associated to any individual condition.

In addition to the advances in machine learning mentioned above, the availability of genomic data (from microarrays and—more recently— whole-genome sequencing) is ever-increasing. This wealth of information has facilitated a vast number of association studies, linking biological variation in countless phenotypes to the underlying genotypes^21^. Some of these studies investigated the genetic basis of ECG-features (e.g. for the PR interval^22^ or the QRS complex^23^), while others sought to determine the impact of genetic variants on the shape of the ECG traces in general^24^ or on a more holistic representation of the cardiac state including the ECG^25^.

In light of these converging developments, we used a previously published convolutional neural network^11^ to predict the “cardiovascular age” of 36,349 participants of the UK biobank (UKB) from their 12-lead ECGs, and performed a GWAS on the difference between predicted and chronological age (i.e. delta age). We found eight loci of genome-wide significance ($p\le 5 \times 10^{-8}$), many of which have been associated with cardiac or muscle development (and in extension with CVD) in the past. Functional and pathway enrichment analyses confirmed this connection to the cardiovascular system. We also explored the association of delta age with specific ECG features, risk factor-derived excess heart age, and the dynamic organism state indicator (DOSI), a complementary biomarker for ageing derived from complete blood count (CBC) data. Overall, our results elucidate the genetic underpinning of this ECG-derived biomarker for cardiovascular age and validate its utility for use in research as well as in the clinic.

## Results

### Predicting age from ECGs in the UK Biobank

We employed a previously described deep learning model trained on patients of the Mayo clinic^11^ to predict the age of 36,349 participants of the UKB from their 12-lead ECGs. On average, individuals were 64 years old, marginally more likely to be women (52%), and had high levels of education (tertiary education for more than 50%). They comprised a relatively healthy cohort (e.g. less than 6% had diagnosed cardiovascular conditions more severe than hypertension), commonly reporting lifestyle choices considered preventive of CVD (e.g. $63\%$ never smoked), and showing predominantly normal ranges for body mass index (BMI), lipids, and blood pressure (Table 1).

**Figure 1:**
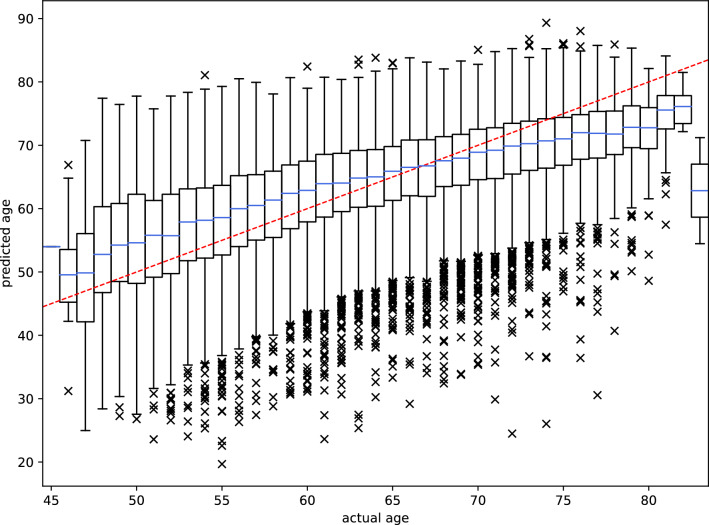
ECG-derived age vs. chronological age for 36,349 participants of the UKB. The Pearson correlation coefficient was 0.53. The red dashed line illustrates a perfect fit. Data is grouped into boxes as the chronological age at the time of recording the ECG was only available as the number of years. Note that ranges and scales are different between the x- and y-axis due to outliers in the predicted age being considerably outside the range of the chronological age in the cohort. Box plot features: blue centre lines, median; box limits, first and third quartile; whiskers, $1.5 \times$ inter-quartile range; markers, outliers.

As the ECGs in the UKB were noisier than those used for training the model originally^11^, an initial signal filtering step was applied prior to prediction. After this pre-processing step, prediction performance on the UKB cohort was comparable to the holdout data set in the original study with a mean absolute error of 6.1 instead of 6.9 years, respectively (Fig. 1). The Pearson correlation coefficient between chronological and predicted age was $\rho$=0.53.

The participants’ chronological ages were then subtracted from the predicted ages to obtain the delta age (median 0.27; interquartile range −4.81–5.15 years). It was strongly associated with certain anthropometric features and cardiovascular conditions (Table 1), consistent with previous studies^3,11,14^. When adjusting for age and sex, tertiary education and physical activity were associated with a lower delta age ($p\le 1 \times 10^{-13}$). BMI, mean arterial pressure (MAP), and low density lipoprotein (LDL), on the other hand, as well as classic cardiovascular risk factors and outcomes, such as frequently drinking alcohol, history of smoking, diagnosed diabetes, hypertension, angina, stroke, or heart attack were associated with higher delta age ($p\le 3 \times 10^{-3}$). These findings were predominantly robust to multivariate analysis when including all mentioned variables in the model (Table 1). Interestingly, men had a lower delta age than women and the negative association with male sex increased when more covariates were taken into account.

Modern ECG machines automatically determine certain human-derived ECG features (e.g., PQ interval, QRS duration) when taking measurements. In the UKB data, many of these features were strongly associated with chronological age, predicted age, or both (Supplementary Table S1). However, only a small fraction of the variance in age could be explained by these human-derived features ($r^2=0.08$ for a linear regression of age on the ECG features). The Pearson correlation coefficient between the age predicted from the ECG features and the chronological age was $\rho$=0.28 (compared to $\rho$=0.53 for the neural network). Interestingly, for the ages predicted by the neural network, this fraction increased almost three-fold ($r^2=0.22$), indicating that the model relies on information retained in these features. This insight has also been shown in a recent study, which found that some features extracted by the convolutional layers of the neural net were strongly correlated with those defined by humans^26^.

**Table 1:** Association of anthropometric features and cardiovascular risk factors in participants of the UKB with delta age.

Covariate	Info ($N_{total}=36,349$)	Adjust for age, sex		Adjust for all	
		Effect size	$P$-value	Effect size	$P$-value
Sex (male)	17607 (48.4%)	−0.56 (−0.71, −0.41)	**4.7e-13**	−1.15 (−1.31, −0.98)	**4.1e-42**
Age	64.25 (±7.57)	−0.37 (−0.38, −0.36)	**0.0e+00**	−0.40 (−0.41, −0.39)	**0.0e+00**
**Education**	–	–	**4.6e-18**	–	**2.4e-06**
Secondary (ref. level)	14437 (40.1%)	–	–	–	–
Tertiary	19186 (53.3%)	−0.69 (−0.85, −0.53)	**1.7e-17**	−0.41 (−0.57, −0.25)	**9.7e-07**
Other	2343 (6.5%)	0.04 (−0.29, 0.36)	0.83	0.01 (−0.33, 0.34)	0.98
**History of health problems:**					
Diabetes	1979 (5.5%)	0.81 (0.47, 1.15)	**2.2e-06**	−0.22 (−0.58, 0.14)	0.23
Hypertension	8419 (23.2%)	1.85 (1.67, 2.04)	**3.7e-88**	0.77 (0.56, 0.97)	**2.1e-13**
Angina	727 (2.0%)	0.88 (0.34, 1.42)	**1.5e-03**	0.11 (−0.48, 0.70)	0.72
Stroke	366 (1.0%)	1.47 (0.71, 2.23)	**1.6e-04**	0.99 (0.20, 1.78)	0.014
Heart attack	524 (1.4%)	1.49 (0.85, 2.13)	**4.6e-06**	1.43 (0.75, 2.12)	**4.1e-05**
**Physiological measurements:**					
BMI	26.62 (±4.25)	0.24 (0.22, 0.25)	**3.6e-149**	0.16 (0.14, 0.18)	**3.8e-55**
MAP	81.11 (±8.89)	0.13 (0.12, 0.14)	**9.9e-173**	0.10 (0.09, 0.11)	**3.0e-80**
LDL [mM]	3.58 (±0.82)	0.15 (0.05, 0.24)	**2.6e-03**	0.03 (−0.07, 0.13)	0.52
**Lifestyle:**					
Smoking	–	–	**6.2e-11**	–	**1.6e-04**
Never / rarely smoked (ref. level)	22477 (62.5%)	–	–	–	–
Active smoker	1300 (3.6%)	0.50 (0.09, 0.92)	0.017	0.41 (−0.01, 0.84)	0.056
Smoked in the past	12212 (33.9%)	0.56 (0.40, 0.72)	**2.3e-11**	0.34 (0.17, 0.51)	**7.9e-05**
Alcohol at least 3x per week	16405 (45.2%)	0.24 (0.09, 0.39)	**2.3e-03**	0.33 (0.17, 0.49)	**6.3e-05**
Days of moderate PA per week	3.72 (±1.87)	−0.16 (−0.20, −0.11)	**8.4e-14**	−0.020 (−0.071, 0.030)	0.43
Days of vigorous PA per week	1.93 (±1.58)	−0.25 (−0.30, −0.20)	**1.1e-24**	−0.16 (−0.22, −0.10)	**2.0e-07**

The “Info” column lists the number of corresponding participants for categorical features (with the percentage of the total population in parentheses) or the mean value for numerical features (with the standard deviation in parentheses). $P$-values and effect sizes in the left double-column are adjusted for age and sex (or only sex for the age-row and vice versa). In the right double-column, the adjustment also includes all other parameters listed in the table. In the “Effect size” columns, values in parentheses denote the lower and upper bounds of the 95% confidence interval. $P$-values smaller than the Bonferroni-corrected threshold ($0.05 / 19 = 0.0026$) are highlighted in bold. BMI, body mass index; MAP, mean arterial pressure; LDL, low-density lipoprotein; PA, physical activity.

**Figure 2:**
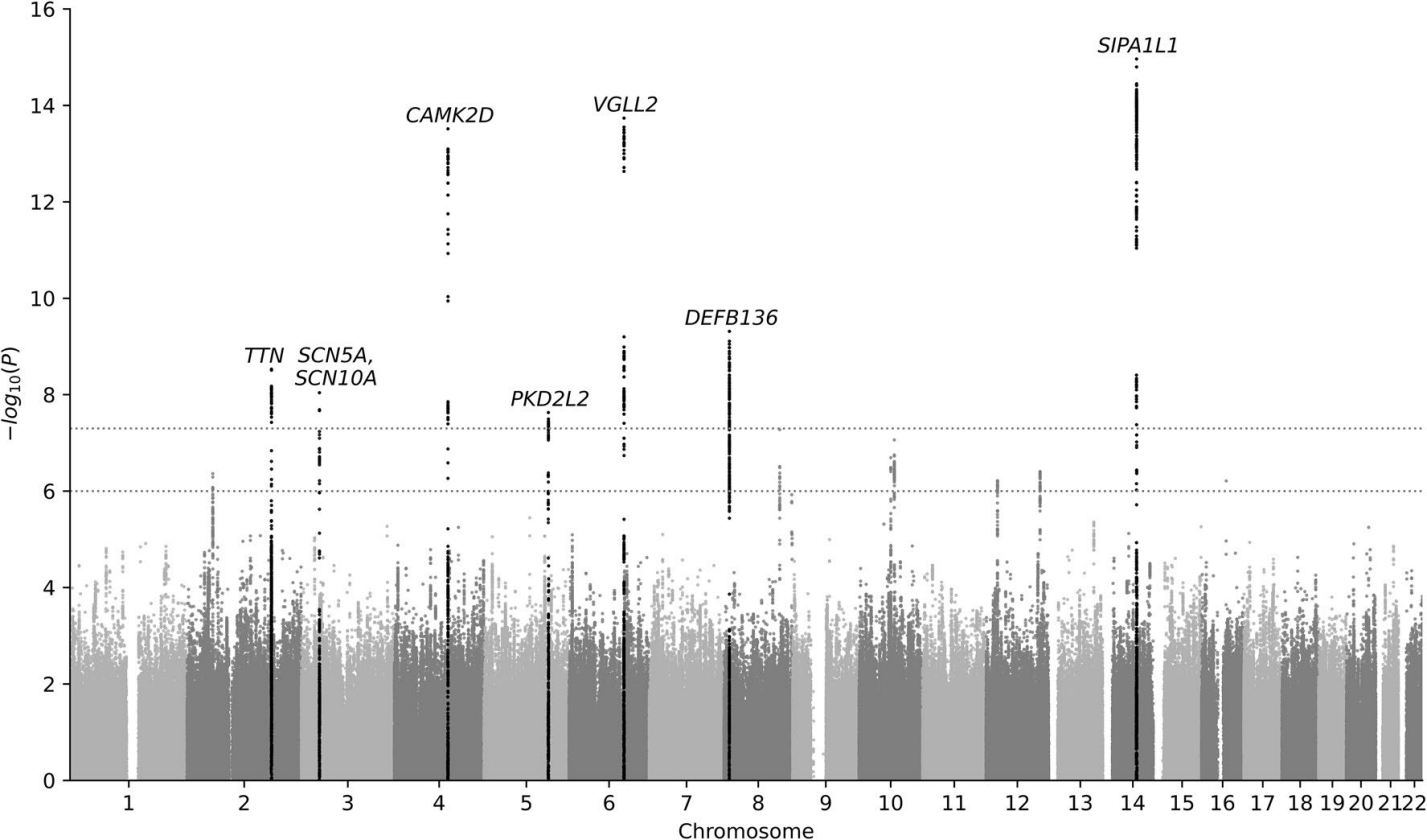
Manhattan plot. Association tests (n=34,432) were adjusted for age, sex, genotyping array, and UKB assessment centre. Horizontal lines mark the thresholds of genome-wide and suggestive significance ($p\le 5 \times 10^{-8}$ and $p\le 1 \times 10^{-6}$, respectively).

### GWAS on delta age

To understand the genetic underpinning of delta age, association tests were performed on $\sim$6.4 million autosomal variants in 34,432 individuals (after filtering and quality control) while adjusting for age, sex, genotyping array, and UKB assessment centre (Fig. 2). This analysis revealed eight loci of genome-wide significance ($p\le 5 \times 10^{-8}$) and another seven loci of suggestive significance ($p\le 1 \times 10^{-6}$; Table 2).

The variants with the strongest association with delta age were detected on chromosome 14 in the gene *SIPA1L1*, which has been linked to ECG features and other cardiac traits according to the GWAS Catalog^27^. Recently, *SIPA1L1* has also been found to be associated with heart trabeculation^28^ and it is involved in the regulation of water transport in the kidney^29^. It might thus have an impact on the cardiovascular system via kidney function or control of blood volume. However, instead of altering *SIPA1L1*, the causal variant in this locus could alternatively affect the expression levels of *RGS6*, which lies $\sim$200 kb downstream. *RGS6* is listed in the GWAS Catalog as associated with systolic blood pressure, heart rate, and heart rate variability, for which there is also mechanistic evidence^30^.

Another strong association signal was found 30–100 kb upstream of *VGLL2* on chromosome 6. *VGLL2* plays a role in the development of skeletal muscle^31^, but, to our knowledge, has not been directly linked to CVD so far. Nonetheless, the GWAS Catalog lists associations with relevant traits like ECG morphology, blood pressure, and atrial fibrillation, but also BMI and waist circumference. Interestingly, *VGLL2* has also been shown to be associated with an age-dependent response to sepsis in the hearts of mice^32^. However, *VGLL2* is not the only protein-coding gene in the region. The next closest ($\sim$100 kb) is *ROS1*, a variant of which has been associated with pathological vascular remodelling^33^.

Variants in *CAMK2D* also showed a strong association with delta age. *CAMK2D* encodes the $\delta$ chain of the ${\hbox {Ca}^{2+}}$/calmodulin-dependent protein kinase II, which phosphorylates (in addition to itself) a wide variety of targets involved in a multitude of cellular functions, including neuroplasticity and memory formation^34^. It also plays a role in cardiac ${\hbox {Ca}^{2+}}$ homeostasis and constitutive activation can lead to CVD and heart failure^35^.

The next notable locus was found on chromosome 8 and many of the variants associated with delta age within this locus have also been associated with essential hypertension in the GWAS Catalog. It was located between a group of three genes for $\beta$-defensins (*DEFB136*, *DEFB135*, *DEFB134* – with *DEFB136* being the closest) and *CTSB*. Being antimicrobial peptides, $\beta$-defensins are an integral part of the innate immune system, but they also have a range of other functions^36^. *CTSB*, located $\sim$50 kb downstream of the variants associated with delta age, codes for cathepsin B, a protease relevant for proteolysis of intracellular proteins as well as constituents of the extracellular matrix^37^. It has been associated with a large number of diseases, including different types of cancer^38^, cardiac remodelling and hypertrophy^39^, as well as atherosclerosis^40^. Interestingly, cathepsin B activity has also been shown to increase with age^41^.

On chromosome 2, variants in *TTN* were associated with delta age. *TTN* codes for the giant protein titin, responsible for passive mechanical properties of muscle (elasticity and stiffness) and sarcomere structure^42^. Mutations in *TTN* (especially when causing truncations) have been linked to dilated cardiomyopathy (DCM)^43^ and the GWAS Catalog mapped a variety of cardiovascular phenotypes and ECG traits to *TTN*, ranging from atrial fibrillation to the PR interval and left ventricular ejection fraction.

*SCN5A* and the neighbouring *SCN10A* (both on chromosome 3) harboured two independent groups of variants at genome-wide significance. Both genes encode subunits of sodium channels (most prevalent in the myocardium^44^ and neurons – including intracardiac ganglia^45^ – respectively). Variants in *SCN5A* have been linked to multiple cardiac disorders and mutations in both genes can cause the arrhythmia-inducing Brugada syndrome^46,47^.

The last locus of genome-wide significance stretched across $\sim$400 kb and six protein-coding genes (*KLHL3*, *HNRNPA0*, *MYOT*, *PKD2L2*, *FAM13B*, and *WNT8A*) on chromosome 5. The gene product of *KLHL3* causes the ubiquitination of substrate proteins and is involved in regulating kidney function^48^. It has been associated with a rare hereditary form of hypertension (familial hyperkalaemic hypertension)^49^ and other forms of congenital heart disease in the past^50^. *FAM13B* encodes a GTPase-activating protein, low expression levels of which have been linked to atrial fibrillation^51^. However, if we assume that there is only one causal variant at this locus, it is most likely to be found in *MYOT*, which codes for myotilin, a component of the Z-disc complex in skeletal and cardiac muscle^52^. Myotilin variants can cause myofibrillar myopathy, which sometimes also affects the heart^53^. We did not find any connections with cardiovascular phenotypes for the other three genes, but the GWAS Catalog lists associations with dysrhythmias and atrial fibrillation across the whole 400 kb-spanning locus and beyond.

The seven extra loci found at suggestive significance ($p\le 1 \times 10^{-6}$) are described in more detail in the Supplementary Results. Most of them were also in the vicinity of genes related to muscle development or the cardiovascular system, but more statistical power (e.g. through larger sample size) will be needed to confirm these associations with delta age.

To assess the robustness of our results, the GWAS was repeated with a more extensive suite of covariates (including history of CVD, exercise, and diet; for details see "Methods" section) and additionally with only those participants that reported a White British ethnic background (Supplementary Fig. S1). All three analyses showed very similar results qualitatively, with a total of 17 loci reaching at least suggestive significance in at least one analysis (Supplementary Table S2).

**Table 2:** Fifteen loci were found to be associated with delta age with at least suggestive significance ($p\le 1 \times 10^{-6}$).

Chr.	Gene	rsID	Pos.	Ref.	Alt.	AF	Effect size	$P$-value
14	*SIPA1L1*	rs35866366	71849185	A	G	0.25	0.52 (0.39, 0.64)	**1.1e-15**
6	*VGLL2*	rs6901720	117510203	G	T	0.47	0.43 (0.32, 0.54)	**2.8e-14**
4	*CAMK2D*	rs35430511	114387138	T	C	0.26	0.49 (0.36, 0.61)	**3.1e-14**
8	*DEFB136*	rs4240678	11802426	C	T	0.40	0.47 (0.32, 0.62)	**4.9e-10**
2	*TTN*	rs11902709	179608207	C	T	0.05	0.78 (0.52, 1.03)	**3.0e-09**
3	*SCN5A*	rs6773331	38684397	A	T	0.98	1.24 (0.82, 1.66)	**9.1e-09**
3	*SCN10A*	rs6801957	38767315	T	C	0.59	−0.32 (−0.43, −0.21)	**2.1e-08**
5	*PKD2L2*	rs10076361	137252940	G	A	0.18	0.41 (0.27, 0.55)	**2.3e-08**
8	*EXT1*	rs57237854	118860126	ATCTTG	A	0.18	0.40 (0.25, 0.54)	5.3e-08
10	*AGAP5*	rs147790633	75447582	T	C	0.14	−0.43 (−0.59, −0.27)	8.7e-08
10	*CTNNA3*	rs72799115	68008504	G	A	0.21	0.35 (0.22, 0.49)	2.0e-07
12	*TBX3*	rs1896329	115357432	C	T	0.69	−0.31 (−0.42, −0.19)	3.9e-07
2	*SPTBN1*	rs1802889	54756740	C	T	0.68	−0.30 (−0.42, −0.19)	4.4e-07
12	*SOX5*	rs12826024	24776799	G	A	0.15	−0.39 (−0.54, −0.24)	6.1e-07
16	*CHD9*	rs75778953	52906677	C	T	0.01	−1.25 (−1.74, −0.76)	6.2e-07

The second column lists the protein-coding gene closest to the respective lead variant. Positions correspond to the GRCh37 human genome assembly^81^. Values in parentheses denote the lower and upper bounds of the 95% confidence interval of the effect size estimate. $P$-values with genome-wide significance ($p\le 5 \times 10^{-8}$) are highlighted in bold. Chr., Chromosome; Pos., Position; Ref., Reference allele; Alt., Alternative allele; AF, frequency of the alternative allele.

### Heritability

The variant-based heritability ($h^2_{\text {g}}$) of delta age was estimated to be $\sim$12%, being robust to adjustment of cardiovascular risk factors ($12.6 \pm 1.7\%$ for regular adjustment and $11.8 \pm 1.8\%$ for extended adjustment). This magnitude is similar to other ECG traits or cardiac phenotypes, such as PR interval (18.2%^22^), long QT syndrome (14.8%^54^), or atrial fibrillation (9.6%^55^). Interestingly, the 15 loci that reached at least suggestive significance only accounted for $\sim$15% of the heritability estimate ($h^2_{\text {g, top15}}=1.8 \pm 0.3\%$ and $1.9 \pm 0.3\%$ for regular and extended adjustment, respectively), indicating that there are likely to be many variants with lower significance that are also relevant.

### Functional analysis and pathway enrichment

As described above, many loci associated with ECG-derived delta age were found in the vicinity of genes involved in cardiac development or have been linked to CVD in the past. Application of the DEPICT enrichment analysis tool^56^ to the 15 loci with at least suggestive significance ($p\le 1 \times 10^{-6}$; see Table 2) revealed that the GO-term with the strongest signal was “intercalated discs”, which are physical connections between cardiomyocytes. The KEGG^57^ pathways with the strongest association were mostly linked to calcium signalling and cardiac afflictions, which was also the case with the Mammalian Phenotype Ontology^58^ gene sets (Supplementary Data 1). We further used DEPICT to test for tissue enrichment. All results with $P$-values smaller than 0.05 were either connective tissues or part of the cardiovascular system (Supplementary Table S3). When including all 179 loci with $p\le 1 \times 10^{-4}$, geneset and tissue enrichment were both dominated by the cardiovascular system (Supplementary Data 2, Supplementary Table S4), reinforcing the robustness of our observations. To confirm these findings with an orthologous method, we additionally employed the gProfiler functional enrichment analysis tool^59^, which also detected a stark overrepresentation of components of the cardiovascular system (Supplementary Table S5). Like the DEPICT analysis, the strength of the enrichment increased when more loci were included (Supplementary Table S6).

### Association of variants in telomere length- and longevity-related genes

Interestingly, genes associated with other forms of biological ageing (e.g. telomere length) were mostly absent from the loci found by our analysis. In order to further investigate this surprising result, we scanned the vicinity of loci discovered by recent GWAS, which had also been performed on the UKB and used longevity^60^ and leukocyte telomere lengths^61^ as phenotypes, for variants associated with delta age. We found that none of the loci associated with longevity and only two of those associated with telomere length (rs12615793 in *ACYP2* and rs12369950 close to *SOX5*) were within one 1 Mb of variants with at least suggestive significance according to our analysis (Supplementary Data 3). In the first case, the lead variant of the locus we discovered was located $\sim$280 kb downstream of rs12615793 and in *SPTBN1*, which is required for heart development^62^. In the second case, rs12369950 was indeed part of the same locus we found to be associated with delta age.

### Further analyses

In order to further investigate the main results described above, we performed statistical tests to detect whether the effects of the genomic variants were mediated via one of the covariates most strongly associated with delta age (BMI, MAP, and diagnosed hypertension), but did not find strong evidence for mediation. Additionally, we ascertained that most of the lead variants have been shown to have a significant impact on the actual shape of the ECG in a recent study^24^. We also calculated the risk factor-based “heart age”^20^ and the whole blood counts-derived DOSI biomarker for ageing^63^ to contrast both with the ECG-derived cardiovascular age. We found that, while the association with delta age was substantial for the “excess” heart age ($p=3.0 \times 10^{-78}$), it was weak for the “excess” DOSI ($p\ge 1.4 \times 10^{-3}$). These findings are described in greater detail in the Supplementary Results.

## Discussion

We used a deep neural network to predict the age of 36,349 individuals in the UKB from their 12-lead ECGs and observed that – similar to what has been shown in other populations^3,11,14^ – the discrepancy to their chronological age was correlated with cardiovascular risk factors like blood pressure, BMI, and smoking status. In addition to these covariates, we also found 15 genetic loci of at least suggestive significance ($p\le 1 \times 10^{-6}$), eight of which reached genome-wide significance ($p\le 5 \times 10^{-8}$), in a GWAS adjusted for age, sex, genotyping array, and UKB assessment centre. We evaluated the robustness of these results by repeating the GWAS with a more extensive set of covariates including past CVD diagnoses and lifestyle variables, such as diet or the amount of physical exercise. We also carried out another round of association tests with only the subset of individuals of European ethnic origin. All three analyses yielded very similar results (Supplementary Table S7). Overall, about 12% of the variation in delta age could be explained by the genomic data, which is comparable to other cardiac phenotypes (e.g. 9.6% for atrial fibrillation^55^).

In order to determine whether the associations of the lead variants with the phenotype were direct and not mediated via an intermediate factor, we performed tests for mediation for the covariates most strongly associated with delta age (MAP, BMI, and diagnosed hypertension). There appeared to be weak mediating effects for some of the variants, but the signal was not strong enough to remain significant after correcting for multiple tests ($p \ge 0.024$). However, some metadata entries in the UKB were recorded a considerable amount of time before the imaging visit when the ECG was taken and some of the covariates might have changed in the intervening period. Because of this limitation and given the large number of (genetic and environmental) factors influencing cardiovascular health and ECG morphology, it is possible that stronger mediating effects might have been missed in the present study. More research will be required in order to disentangle the network of interactions between genetic and non-genetic variables affecting cardiovascular age and its impact on the ECG.

Most of the loci discovered in our GWAS analysis have either been associated with CVD in the past or were located in the vicinity of genes involved in cardiovascular function. Functional analyses with the DEPICT enrichment analysis tool^56^ found significant over-representation of gene sets related to cardiac and muscle development as well as of genes expressed in the corresponding tissues. These associations were confirmed with an alternative method (gProfiler^59^) and grew stronger and more robust when variants with weaker association with delta age were included in the analysis (i.e. when using $P$-value cutoffs of $p\le 1 \times 10^{-5}$ or $p\le 1 \times 10^{-4}$). Similarly, only a small fraction ($\sim$15%) of the heritability we found could be explained by the 15 top loci. Together, these two findings suggest that many of the variants with only moderate significance might also be potential components of the genetic basis of delta age, but larger studies will be needed to verify their signal.

In addition to their links to CVD, the lead variants in most loci of genome-wide significance have also been associated with the actual shape of the ECG in a recent study^24^. This is a promising sign as it might help to illuminate the “black box” character of the neural network used for age prediction. In general, the knowledge about the effects of age on the ECG and the impact of genetic variants should be combined in order to aid in the interpretation of results produced by opaque deep learning models in the medical domain.

In addition to the relatively large sample sizes possible with easily obtainable phenotypes like the ECG, another interesting aspect of using metrics like delta age (or the shape of the ECG as done in^24^) in association studies is that they provide a relatively “dense” signal compared to binary variables (e.g. the absence or presence of a certain type of CVD – especially when the condition is rare and / or easily misdiagnosed). Similarly, using the output of artificial intelligence (AI) models trained on diagnosing such diseases from the ECG as phenotypes might improve statistical power as their predictions need not be binary (i.e. they can – to a certain extent – quantify the severity of the condition) and they might detect diseased cases that were undiagnosed in the original data.

Several different biomarkers for ageing have been proposed in the last two decades, with telomere length and the epigenetic clock arguably receiving the most attention. Despite each being a good predictor for mortality, these metrics were shown to only correlate weakly with each other, implying that they are governed by different aspects of the mechanisms of ageing^8,9^. We observed something similar as we did not find a strong association of variants previously linked to ageing^60^ or telomere length^61^ with delta age. We also calculated the DOSI, a blood counts-derived marker for biological ageing and physiological resilience^63^, for our cohort and – as opposed to the risk factor-derived “excess” heart age – correlation of the “excess” DOSI with delta age was inconclusive. More research relating different markers of biological ageing with delta age is needed, but the available evidence suggests that genetic variants associated with more general forms of ageing (e.g. in *APOE*, *FOXO3*, *TERT*, *LMNA*) have little impact on cardiovascular age compared to genes involved in the development and function of the cardiovascular system itself.

Viewed in their entirety, our findings corroborate that the ECG-derived age reflects the physiological state of the heart and that it can be used to assess cardiovascular ageing and health. Interestingly, for two of the loci with the strongest association with delta age (*SIPA1L1* and *VGLL2*), the connection to cardiovascular phenotypes in the literature was not as clear as for many others. They therefore represent promising targets for deeper mechanistic investigation in future work. Additionally, efforts on fine-mapping will be needed to identify individual causal variants and also to confirm relevant genes since variants in linkage disequilibrium with the lead variant spanned hundreds of kilobases for some of the loci found in this study. This raises the opportunity of narrowing down the range of potential causal variants with association studies in populations of non-European ancestry.

Our work shows that genetic factors underlying cardiovascular ageing and its effect on the ECG should be incorporated into prediction models in order to improve their accuracy and interpretability. In a future of personalised medicine with readily available genomic information, the non-invasive ECG (including from wearable devices), combined with an easily obtainable measure of ECG-derived delta age, will be a valuable instrument in the clinicians’ toolkit for assessing heart health at routine examinations and monitoring treatment outcomes. Moreover, resources like the UKB, hosting an ever-increasing wealth of genomic, epigenetic, and transcriptomic data, will facilitate better comparisons as well as deeper understanding of the individual biomarkers for ageing, their underlying mechanisms, and how they complement one another. Ultimately, large-scale analysis of such data, combined with AI methodologies, will translate patient-level genomic and ECG information into preventative medicine and public health measures, leading to earlier detection of CVD and a longer healthspan.

## Methods

### Study population

This work has been conducted using data from the UKB, which recruited 500,000+ people aged between 40 and 69 years in 2006–2010 from across the United Kingdom^64^. With their informed consent, they provided detailed information about their lifestyle, had physical measures taken as well as blood, urine and saliva samples collected and stored for future analysis. We used the 10-second 12-lead ECG traces and CVD-related metadata of 37,520 participants. The ECGs were recorded during the first imaging visit (after 2014) and the metadata questionnaires were completed during the initial and first repeat assessment visits (2006–2010 and 2012–2013, respectively). All analyses were performed in accordance with relevant guidelines and regulations posed by the UKB and approved by the London School of Hygiene & Tropical Medicine ethics committee. The UKB project application reference was 54050 (www.ukbiobank.ac.uk).

### Deep learning model, ECG pre-processing, and age prediction

The architecture and training procedure of the deep learning model used in this study are described in more detail in the Supplementary Methods and in the original publication^11^. In brief, 499,727 10-second 12-lead ECGs of patients of the Mayo clinic were used to train a convolutional neural network to predict patient age and a holdout dataset of 275,056 patients was used for testing model performance. The neural network is comprised of eight convolutional blocks in the temporal dimension, the outputs of which are combined in a single convolutional layer across the “spatial” dimension (i.e. across the 12 leads of the ECGs) with max-pooling. This is followed by two fully connected layers before being passed to the linear output layer producing the age prediction.

Due to the ECGs in the UKB being noisier than the training data, they had to undergo a filtering step prior to prediction. This was achieved using a four-pole Butterworth filter allowing frequencies from 0.5 to 100 Hz to pass. After pre-processing, ECG-derived age was predicted for 36,349 individuals in the UKB.

### Metadata processing

Whenever multiple measurements of a relevant variable were available for a given sample, the mean or the value with the smallest time gap to the ECG recording was used for continuous and categorical data, respectively. MAP was calculated from systolic (SBP) and diastolic blood pressure (DBP) measurements using the equation (SBP + 2 $\cdot$ DBP) / 3. These MAP values were then averaged with the MAP measurements derived from Pulse Wave Analysis to give the final values. The UKB contains a host of diet variables ranging from the amount of raw vegetables eaten per day to the type of fat used for cooking. We performed principal component analysis (PCA) on a selection of 24 of these variables and included the first three principal components (accounting for $\sim$25% of the total variation) as covariates in the GWAS with extended adjustment (see below).

### Association testing

Pre-processing of genotype data and association testing were carried out using PLINK (v. 2.00)^65^. For quality control, we removed variants that either: (1) were missing in more than 1% of samples, (2) had a minor allele frequency less than 1%, (3) were not in Hardy-Weinberg Equilibrium ($P < 1\times 10^{-6}$), or (4) had an imputation score below 0.8. Samples with more than 2% missing genotypes or that were outside of three standard deviations from the mean heterozygosity were dropped. Additionally, one sample from each closely related pair (first or second degree relations as determined by KING robust kinship inference^66^) was removed. The dimension of the final genotype matrix was 34,432 samples times 6,357,764 autosomal variants. PCA^67^ was performed on this matrix and the first 10 principal components were retained for use as covariates in the association tests.

In total, four GWAS with delta age as phenotype were carried out. The main analysis included all participants remaining after filtering and adjusted for age, sex, genotyping array, and UKB assessment centre. Additionally, in order to assess the robustness of the results, the association tests were repeated with an extended set of covariates: education (secondary, tertiary, other); smoking status (current smoker, past smoker, never / rarely smoked); alcohol consumption three or more times per week; having been diagnosed with diabetes, hypertension, angina, stroke, or heart attack in the past; BMI; MAP; LDL concentration; days of moderate exercise per week; days of vigorous exercise per week; and three principal components derived from a PCA of 24 diet variables available in the UKB. Both analyses were then repeated with the subset of participants with white British as ethnic background ($N=31,971$).

### Heritability estimation and pathway enrichment analysis

The variant-based heritability of delta age was estimated using GREML-LDMS^68^ implemented in GCTA (v. 1.93.2)^69^ while stratifying the variants based on linkage disequilibrium (four bins) and minor allele frequency (MAF) (two bins with $\text {MAF}=0.05$ as boundary). The analysis was carried out with both sets of covariates and later repeated with the subsets of variants found within the 15 loci of at least suggestive significance in order to also calculate the heritability of the top hits found by the GWAS. Genomic position ranges of the individual loci were calculated as part of the DEPICT workflow. DEPICT^56^ and gProfiler^59^ were used for pathway and tissue enrichment analyses. DEPICT was run on the GWAS summary statistics with $p=1 \times 10^{-6}$ and $p=1 \times 10^{-4}$ as thresholds. It uses PLINK internally to determine independent loci based on the $P$-value threshold and a 500 kb clumping window before testing for gene set and tissue enrichment relying on data from the following databases: Gene Ontology^70^, KEGG^57^, Reactome^71^, InWeb^72^, Mouse Genome Database^73^, and Gene Expression Omnibus^74^. The coordinates of the loci found by DEPICT were additionally pasted into the gProfiler web tool, which tested for enrichment based on the Gene Ontology, KEGG, Reactome, WikiPathways^75^, TRANSFAC^76^, miRTarBase^77^, Human Protein Atlas^78^, CORUM^79^, and Human Phenotype Ontology^80^ databases.

## Supplementary Information

Supplementary Information 1.

Supplementary Information 2.

Supplementary Information 3.

Supplementary Information 4.

Supplementary Information 5.

## Data Availability

All data is available from the UKB (www.ukbiobank.ac.uk).
