# Supplementary material for: Deep learning-derived cardiovascular age shares a genetic basis with other cardiac phenotypes
Source: Sci Rep. 2022 Dec 31;12:22625. doi: 10.1038/s41598-022-27254-z (PMC9805465; doi:10.1038/s41598-022-27254-z)
Supplement: Supplementary file 1 — Supplementary Information 1. [file 41598_2022_27254_MOESM1_ESM.pdf]

SUPPLEMENTARY INFORMATION

—

Deep learning-derived cardiovascular age shares a genetic basis with other cardiac phenotypes

Julian Libiseller-Egger[1], Jody E. Phelan[1], Zachi I. Attia[2], Ernest Diez Benavente[1,3], Susana Campino[1], Paul A. Friedman[2], Francisco Lopez-Jimenez[2], David A. Leon[4,5], Taane G. Clark[1,4,*]

December 6, 2022

1 Faculty of Infectious and Tropical Diseases, London School of Hygiene & Tropical Medicine, London, UK
2 Department of Cardiovascular Medicine, Mayo Clinic College of Medicine, Rochester, MN, USA
3 Laboratory of Experimental Cardiology, University Medical Center Utrecht, Utrecht, Netherlands
4 Faculty of Epidemiology and Population Health, London School of Hygiene & Tropical Medicine, London, UK
5 Department of Community Medicine, UiT the Arctic University of Norway, Tromsø, Norway
***** Corresponding author

Supplementary Methods

Convolutional neural network for predicting age from the 12-lead ECG

Training and testing data

In total, 10-second 12-lead electrocardiograms (ECGs) of 774,783 patients (not more than one ECG per patient) from the Mayo Clinic digital data vault were used during the creation and initial testing of the neural network used in this study [1]. 275,056 of the ECGs were kept as holdout test set (i.e. these data were not seen by the model during training). One quarter of the remaining 499,727 ECGs were put aside for internal validation while the other 75% were used for training.

Neural network architecture

The neural network contains nine convolutional blocks (eight in the temporal direction – i.e. looking at a short segment of a single ECG lead at a time – and one in the "spatial" direction for combining information from all leads). Each block is made up of a 1-D convolutional layer with kernel sizes between three and seven and 16 to 64 filters, a batch normalisation layer [2] feeding into ReLU activation, and a max-pooling layer kernel sizes of two or four. The output of the final convolutional block is passed through two fully connected layers with 128 and 64 neurons, respectively, before age is predicted by the final linear output layer.

Hyper parameters

Backpropagation was performed using the Adam optimiser [3] with mean squared error as loss function. Learning rates between 3×10^{-3} and 10^{-5} as well as batch sizes between 16 and 64 were tested during hyperparameter optimisation (the values finally chosen were 3×10^{-4} and 64, respectively).

For more details on model creation and validation, see [1].

Supplementary Results

GWAS results of suggestive significance

The genome-wide association study (GWAS) with regular adjustment found eight loci associated with delta age at genome-wide significance ($p \leq 5 \times 10^{-8}$; for details see Results section in the main text) and seven loci at suggestive significance ($p \leq 1 \times 10^{-6}$) which are described in more detail below.

EXT1 on chromosome 8 was just short of reaching genome-wide significance. It encodes a glycosyltransferase involved in the synthesis of heparan sulfate, a component of the extracellular matrix with a wide array of functions [4], including a crucial role in early development [5]. *EXT1* itself has also been linked to the development of the heart [6]. On chromosome 10, two loci with lead variants in *AGAP5* and *CTNNA3* were identified. *CTNNA3* codes for αT-catenin, a component of cell junctions called intercalated discs which mechanically connect myocytes [7]. The other locus comprised a large number of variants in linkage disequilibrium, spanning ~200 kb and several genes (*MYOZ1*, *SYNPO2L*, *AGAP5*, *SEC24C*, *FUT11*, *CHCHD1*, *ZSWIM8*, *AC022400.5*, *NDST2*, and *CAMK2G*). Out of these, *MYOZ1* and *SYNPO2L* are relevant for cardiac processes. *MYOZ1* binds to Z-disc proteins, is involved in calcineurin signalling [8], and mutations in it have been linked to atrial fibrillation [9] as well as heart failure [10]. Similarly, *SYNPO2L* also interacts with Z-disc proteins and it too has been shown to be associated with atrial fibrillation [11, 12]. Moreover, the GWAS Catalog lists associations for *SYNPO2L* with ECG morphology, hypertrophic cardiomyopathy, and blood pressure, among other cardiac phenotypes.

Variants in *SOX5* on chromosome 12 were also associated with delta age at suggestive significance, with links to ECG morphology, atrial fibrillation, and blood pressure, in the GWAS Catalog. *SOX5* is involved in heart development in *D. melanogaster* [13] and has been found to be associated with dilated cardiomyopathy (DCM) [14]. The second locus on chromosome 12 was found ~225 kb upstream of *TBX3*, which was the closest protein-coding gene. It is a transcription factor involved in the development of multiple tissues, including within the heart [15]. Variants upstream of *TBX3* have been associated with pacemaker cell function [16]. The GWAS Catalog also lists associations with multiple cardiac phenotypes for *TBX3*, including blood pressure, ECG morphology, PR interval, heart failure, and others.

In addition to *TTN*, which reached genome-wide significance, another locus on chromosome 2 was found to be associated with delta age. *SPTBN1* encodes II spectrin, which is required for normal cardiac development as well as excitability [17]. In the GWAS Catalog it has been linked to a long list of phenotypes, some of which relate to the cardiovascular system (e.g. ECG morphology, PR interval, or glomerular filtration rate).

The final locus reaching suggestive significance was located on chromosome 16 with the closest protein-coding gene being *CHD9*, a chromatin remodelling protein. We did not find a direct connection of *CHD9* to heart development or cardiovascular disease (CVD) and no cardiac phenotypes were linked in the GWAS Catalog. Also, only a single rare (minor allele frequency (MAF) =1.2%) variant crossed the significance threshold at this locus and it was not in strong linkage disequilibrium with any other variants in the region. Moreover, it lost suggestive significance in the dataset with only white British ethnic background (**Supplementary Table 2**). Taken together, these findings might indicate a spurious association. Thus, in order to confirm the signal at this locus, future studies with larger and more ethnically diverse datasets are needed.

Mediation analyses

Whilst most GWAS results were robust with respect to the differing levels of adjustment for potential confounders, there were some lead variants with considerable quantitative (but not qualitative) differences in significance. This observation may arise from the variants affecting delta age (at least partially) via mediation of one or more of the covariates (e.g. a variant not influencing delta age directly but rather blood pressure which itself then impacts ECG-derived age). Therefore, we ran mediation analyses for mean arterial pressure (MAP), body mass index (BMI), and hypertension as individual mediators while adjusting for the regular covariates. They were carried out using the Pingouin Python package [18] and with 5,000 bootstrap iterations per test. The results suggested minor mediating effects for some variant-mediator combinations, but none of them remained significant when

taking multiple testing into account ($p_{min} = 0.024$; **Supplementary Data 4**), indicating that the impact of the genomic variants on delta age was indeed direct.

Impact of lead variants on ECG morphology

In a recent study, 3-lead ECGs were aligned and averaged to generate one representative time trace of a single heartbeat for more than 70,000 participants in the UK biobank (UKB) [19]. The aligned signal was then split into 500 data points per individual and 500 GWAS were performed to identify variants associated with each of these subsections of the ECG. This resulted in 300+ significant loci, many of which had not been reported in a cardiac context before, and revealed novel insights into diseases like DCM and early repolarisation. A webtool (`www.ecgenetics.org`) was developed for browsing the results and to look up the impact of a given variant on the shape of the ECG. We used this tool to assess our GWAS results. Seven out of eight loci with genome-wide significant association with delta age also exhibited genome-wide significance ($p = 1 \times 10^{-10}$ when correcting for 500 tests) for at least one section of the ECG in the study of Verweij *et al.* [19] (**Supplementary Table 2**). Out of all lead variants found by our analyses, those in *SCN5A*, *SCN10A*, and *TBX3* were shown to have the strongest impact on ECG morphology, all three of which are connected to the electrophysiology of the heart (for details see above and the Results section in the main text).

Comparison with risk factor-derived "heart age"

The "heart age" of a person is a function of a list of risk factors and is defined as the age of somebody who has the same risk of CVD as the original person, but for whom all these risk factors are at normal levels [20]. Thus, with the exception of being derived exclusively from risk factors, the "excess heart age" (the difference between heart age and chronological age) is conceptually similar to delta age. In order to calculate the heart age of the individuals in our cohort, we extracted data on the relevant risk factors from the UKB and adapted the source code (available at `https://github.com/Antbits/heartage`) of an online tool [21] of the National Health Service, which relies on a model for heart age fitted on a British population [22]. We then subtracted the heart age from the chronological age of the participants to determine the excess heart age and compared it to delta age. Overall, excess heart age was strongly correlated with delta age ($p = 3.0 \times 10^{-78}$ when adjusting for chronological age; delta age increased by 0.108–0.134 for each year of excess heart age). This was not surprising as many of the the risk factors comprising the heart age were individually correlated with delta age (**Table 1**), but it reaffirms the connection of structural / functional features of the heart – as picked up by the ECG-derived delta age – with cardiovascular risk factors and lifestyle choices.

Association with the Dynamic Organism State Indicator

The relevant blood count data were extracted and used to determine the dynamic organism state indicator (DOSI) metric [23]. Then, the "excess" DOSI was calculated by fitting cubic splines to the DOSI against age for each sex and subsequently subtracting the value of the spline for the corresponding age from the individual DOSI values. When adjusting for age, sex, and the UKB assessment centre, we found neither the DOSI nor the excess DOSI to be associated with delta age. However, when including all other covariates which were also corrected for in the GWAS with extended adjustment, there was a weak association with both metrics ($p \leq 4 \times 10^{-3}$; see **Supplementary Table 7**).

GWAS on abnormal versus normal ECGs

Many ECG machines give automatic diagnoses when taking a measurement, including reporting a "normal" or "abnormal" status. Age predictions on such "abnormal" ECGs were consistently higher on average compared to "normal" ECGs (**Supplementary Figure 2**). In addition to the delta age analysis, we ran two extra GWAS (using regular and extended adjustment like for the main analysis) on the abnormal-vs-normal phenotype to see if we could replicate the findings of a recent study done on a relatively small Chinese cohort ($n = 1006$) [24]. However, no variants reached genome-wide significance ($p \leq 5 \times 10^{-8}$) and the variants reaching suggestive significance ($p = 1 \times 10^{-6}$) adjusting

for either set of covariates were different from those reported by the study (**Supplementary Figure 3**, **Supplementary Table 8**).

Supplementary Figures

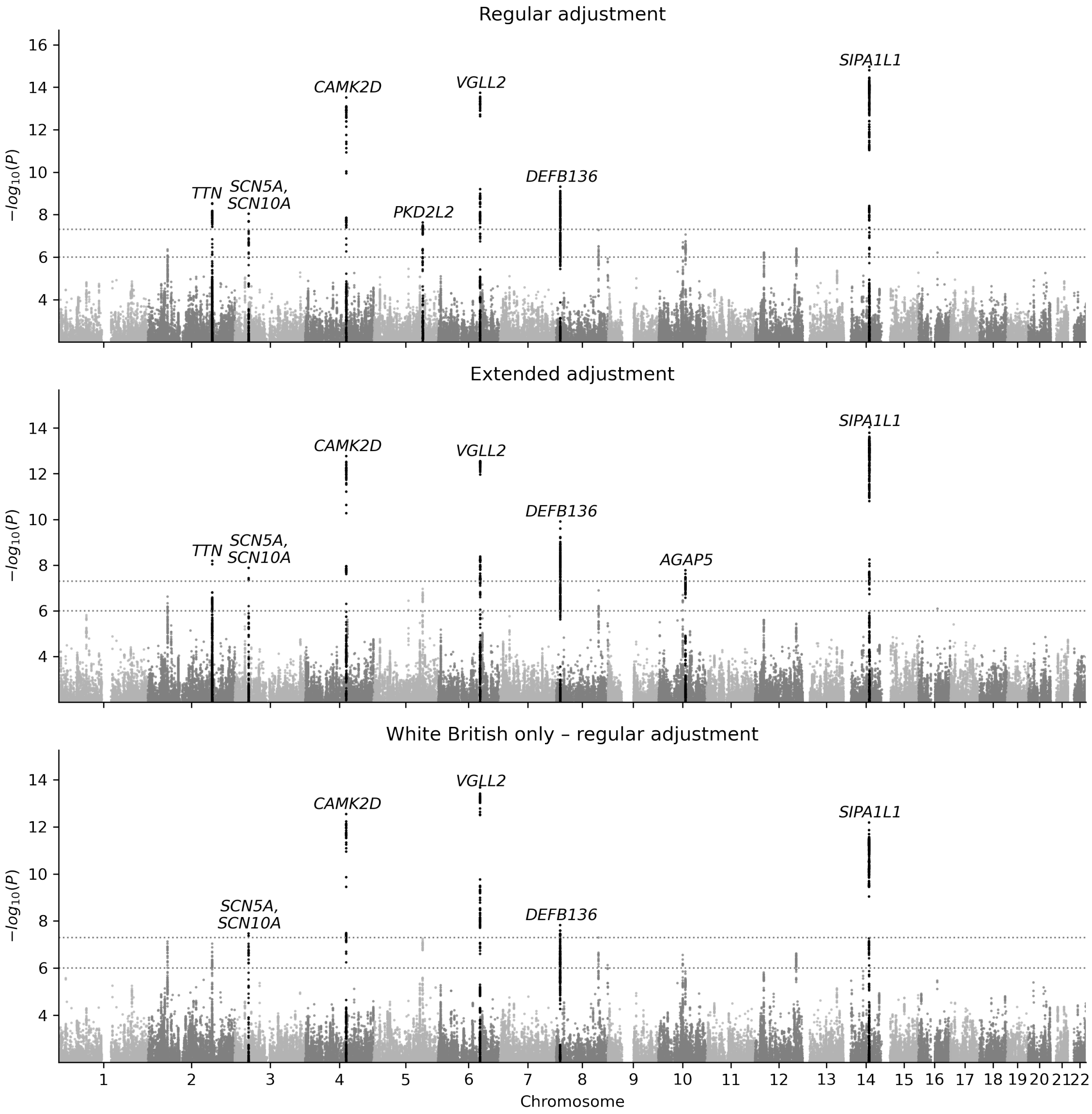

Supplementary Figure 1: Manhattan plots for contrasting the GWAS results using "regular" adjustment (age, sex, genotyping array, assessment centre) and "extended" adjustment (see Methods section for a full list of covariates) of the whole dataset as well as a subset including only participants with an ethnic background of "White British".

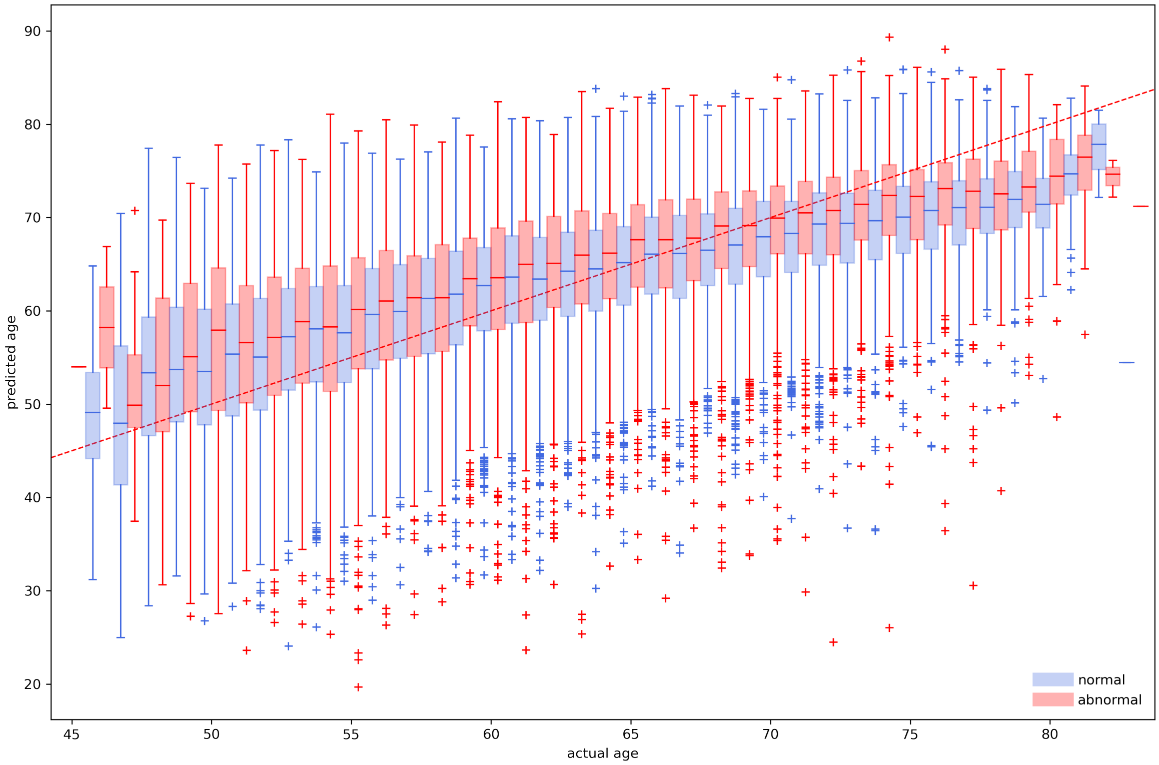

Supplementary Figure 2: Age predicted from 12-lead ECGs by a deep convolutional neural network vs. chronological age for 36,349 participants of the UKB. Predictions from "abnormal" ECG s (as automatically determined by the ECG machine) are shown in red; predictions from other ECGs in blue.

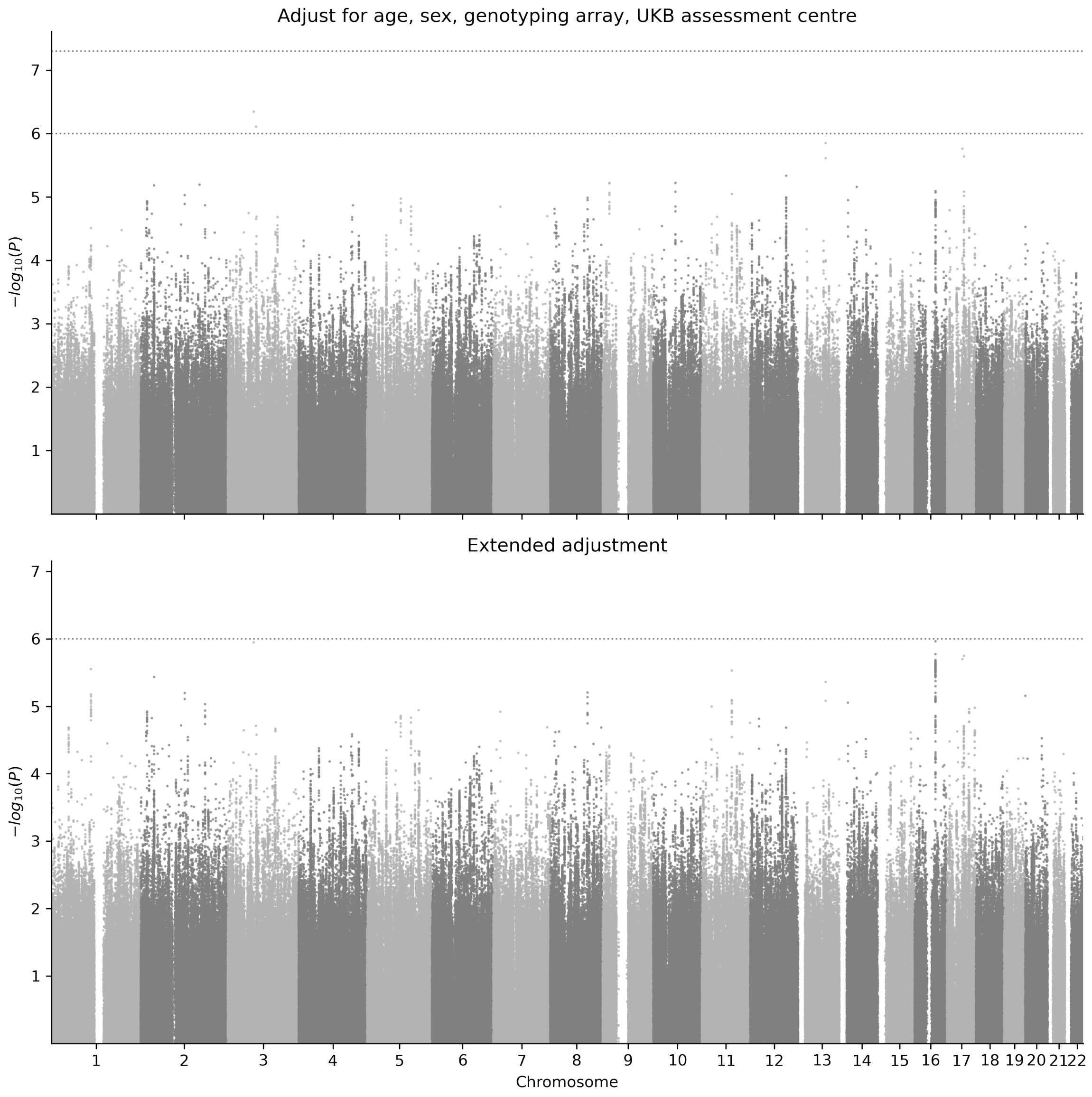

Supplementary Figure 3: Manhattan plots of the association tests for "regular" (age, sex, genotyping array, UKB assessment centre) and extended (see Methods for the full list of covariates) adjustment with abnormal vs. normal ECG as binary phenotype. Dotted lines show the thresholds for genome-wide and suggestive significance ($p = 5 \times 10^{-8}$ and $p = 1 \times 10^{-6}$, respectively).

Supplementary Tables

Supplementary Table 1: Association of human-defined ECG features with chronological and ECG-derived age.

		Univariate		Multivariate (without age)	
		Effect size	*P*-value	Effect size	*P*-value
Ventricular rate	Age	0.49 (0.41, 0.57)	**2.1e-34**	0.24 (0.16, 0.32)	**3.6e-9**
	Pred. age	0.51 (0.42, 0.60)	**2.0e-27**	-0.06 (-0.16, 0.03)	0.19
P duration	Age	0.31 (0.23, 0.39)	**6.2e-14**	0.19 (0.12, 0.27)	**1.0e-6**
	Pred. age	0.55 (0.46, 0.64)	**1.1e-31**	0.49 (0.40, 0.58)	**7.7e-26**
PP interval	Age	-0.57 (-0.66, -0.47)	**5.1e-30**	-0.27 (-0.37, -0.17)	**4.7e-8**
	Pred. age	-0.63 (-0.75, -0.52)	**6.3e-27**	-0.03 (-0.15, 0.09)	0.64
PQ interval	Age	1.27 (1.17, 1.36)	**6.2e-144**	1.07 (0.97, 1.16)	**9.7e-108**
	Pred. age	2.36 (2.25, 2.47)	**0.0**	2.32 (2.21, 2.43)	**0.0**
QRS num	Age	0.39 (0.29, 0.49)	**1.1e-14**	0.11 (0.01, 0.21)	0.025
	Pred. age	0.37 (0.25, 0.48)	**6.4e-10**	-0.23 (-0.36, -0.11)	**1.5e-4**
QRS duration	Age	0.68 (0.60, 0.76)	**2.7e-63**	0.41 (0.33, 0.49)	**9.3e-24**
	Pred. age	0.61 (0.52, 0.71)	**1.5e-39**	0.46 (0.36, 0.55)	**9.4e-21**
QT interval	Age	0.13 (0.03, 0.23)	9.8e-3	0.34 (0.24, 0.43)	**7.2e-12**
	Pred. age	0.51 (0.39, 0.63)	**1.2e-17**	0.87 (0.75, 0.99)	**2.9e-47**
QTC interval	Age	0.96 (0.86, 1.07)	**6.0e-73**	0.81 (0.71, 0.92)	**2.2e-52**
	Pred. age	1.57 (1.45, 1.70)	**4.8e-138**	1.14 (1.01, 1.26)	**1.1e-67**
RR interval	Age	-0.50 (-0.60, -0.41)	**1.9e-24**	-0.22 (-0.32, -0.12)	**1.2e-5**
	Pred. age	-0.55 (-0.67, -0.44)	**3.5e-21**	0.08 (-0.04, 0.20)	0.20
P axis	Age	0.62 (0.52, 0.71)	**6.4e-35**	0.61 (0.52, 0.71)	**8.5e-37**
	Pred. age	0.89 (0.78, 1.00)	**8.9e-53**	1.11 (0.99, 1.22)	**1.6e-80**
R axis	Age	-1.21 (-1.30, -1.11)	**4.2e-137**	-0.95 (-1.05, -0.86)	**1.8e-86**
	Pred. age	-3.19 (-3.29, -3.08)	**0.0**	-2.87 (-2.98, -2.76)	**0.0**
T axis	Age	0.33 (0.23, 0.42)	**2.4e-11**	0.31 (0.22, 0.40)	**1.1e-10**
	Pred. age	-0.41 (-0.53, -0.30)	**1.4e-12**	-0.30 (-0.41, -0.18)	**3.4e-7**

To allow for comparable effect sizes, the ECG features were divided by their standard deviations prior to running the regression tests. Linear models used to generate the results shown in the 'Multivariate' column included all covariates listed for "extended adjustment" in the Methods section except for age. Values in parentheses denote the lower and upper bounds of the 95% confidence interval. *P*-values smaller than the Bonferroni-corrected threshold (0.05/24 = 0.0021) are highlighted in bold.

Supplementary Table 2: Loci found by our GWAS analyses to be associated with delta age and corresponding *P*-values of association with ECG morphology as determined by Verweij *et al.* [19].

Chr.	Gene	rsID	Pos.	Ref.	Alt.	AF	Type	Effect size	*P*-value (assoc. with delta age)	*P*-value (assoc. with ECG morph.)
2	*SPTBN1*	rs1802889	54,756,740	C	T	0.68	regular	-0.30 (-0.42, -0.19)	4.4e-07*	**2.3e-13**
							extended	-0.32 (-0.43, -0.20)	2.4e-07*	
							white British	-0.34 (-0.46, -0.21)	7.5e-08*	
	TTN	rs12464215	179,690,968	G	T	0.46	regular	0.31 (0.20, 0.42)	**2.9e-08**	**8.0e-25**
							extended	0.33 (0.22, 0.44)	**6.5e-09**	
							white British	0.31 (0.20, 0.42)	9.2e-08*	
3	*SCN5A*	rs6773331	38,684,397	A	T	0.98	regular	1.24 (0.82, 1.66)	**9.1e-09**	**5.7e-71**
							extended	1.24 (0.81, 1.67)	**1.3e-08**	
							white British	1.22 (0.79, 1.66)	**3.4e-08**	
	SCN10A	rs6801957	38,767,315	T	C	0.59	regular	-0.32 (-0.43, -0.21)	**2.1e-08**	**2.8e-133**
							extended	-0.29 (-0.40, -0.17)	6.3e-07*	
							white British	-0.32 (-0.44, -0.21)	**4.3e-08**	
4	*CAMK2D*	rs35430511	114,387,138	T	C	0.26	regular	0.49 (0.36, 0.61)	**3.1e-14**	**2.9e-22**
							extended	0.48 (0.35, 0.61)	**1.7e-13**	
							white British	0.48 (0.35, 0.61)	**2.9e-13**	
5	*RGMB*	rs59839451	97,514,975	G	T	0.02	regular	-0.92 (-1.31, -0.53)	3.6e-06	7.8e-03
							extended	-1.03 (-1.42, -0.63)	3.7e-07*	
							white British	-0.87 (-1.27, -0.46)	2.5e-05	
	PKD2L2	rs10076361	137,252,940	G	A	0.18	regular	0.41 (0.27, 0.55)	**2.3e-08**	1.6e-03
							extended	0.40 (0.25, 0.54)	1.1e-07*	
							white British	0.41 (0.26, 0.56)	5.7e-08*	
6	*VGLL2*	rs6901720	117,510,203	G	T	0.47	regular	0.43 (0.32, 0.54)	**2.8e-14**	**1.0e-10**
							extended	0.42 (0.31, 0.53)	**2.9e-13**	
							white British	0.44 (0.33, 0.55)	**4.5e-14**	
8	*DEFB136*	rs4240678	11,802,426	C	T	0.40	regular	0.47 (0.32, 0.62)	**4.9e-10**	**1.7e-18**
							extended	0.49 (0.34, 0.64)	**1.2e-10**	
							white British	0.44 (0.29, 0.59)	**1.5e-08**	
	EXT1	rs57237854	118,860,126	ATCTTG	A	0.18	regular	0.40 (0.25, 0.54)	5.3e-08*	5.9e-07
							extended	0.39 (0.25, 0.54)	1.3e-07*	
							white British	0.41 (0.26, 0.56)	5.1e-08*	
	ZC3H3	rs2294117	144,520,147	G	A	0.25	regular	0.31 (0.19, 0.44)	1.2e-06	1.2e-02
							extended	0.30 (0.18, 0.43)	3.6e-06	
							white British	0.33 (0.20, 0.46)	7.5e-07*	
10	*CTNNA3*	rs72799115	68,008,504	G	A	0.21	regular	0.35 (0.22, 0.49)	2.0e-07*	8.9e-07
							extended	0.36 (0.22, 0.49)	2.1e-07*	
							white British	0.36 (0.22, 0.50)	4.3e-07*	
	AGAP5	rs147790633	75,447,582	T	C	0.14	regular	-0.43 (-0.59, -0.27)	8.7e-08*	**3.5e-24**
							extended	-0.46 (-0.62, -0.30)	**1.7e-08**	
							white British	-0.40 (-0.57, -0.24)	1.4e-06	
12	*SOX5*	rs12826024	24,776,799	G	A	0.15	regular	-0.39 (-0.54, -0.24)	6.1e-07*	**1.7e-21**
							extended	-0.37 (-0.53, -0.22)	2.5e-06	
							white British	-0.38 (-0.54, -0.22)	2.4e-06	
	TBX3	rs1896329	115,357,432	C	T	0.69	regular	-0.31 (-0.42, -0.19)	3.9e-07*	**6.2e-110**
							extended	-0.28 (-0.40, -0.16)	5.4e-06	
							white British	-0.32 (-0.45, -0.20)	2.7e-07*	
14	*SIPA1L1*	rs35866366	71,849,185	A	G	0.25	regular	0.52 (0.39, 0.64)	**1.1e-15**	**2.2e-13**
							extended	0.51 (0.38, 0.63)	**9.3e-15**	
							white British	0.48 (0.35, 0.61)	**6.6e-13**	
16	*CHD9*	rs75778953	52,906,677	C	T	0.01	regular	-1.25 (-1.74, -0.76)	6.2e-07*	2.3e-03
							extended	-1.25 (-1.75, -0.75)	8.1e-07*	
							white British	-1.19 (-1.70, -0.69)	3.4e-06	

This table lists all loci and lead variants which were associated with delta age with at least suggestive significance ($p \leq 1 \times 10^{-6}$) in at least one GWAS run (i.e. with regular adjustment, extended adjustment, or regular adjustment including only participants with White British ethnic background; regular adjustment includes age, sex, genotyping array, and UKB assessment centre; see Methods for a list of covariates included in extended adjustment). For some loci, not all GWAS runs featured the same lead variant (i.e. variant with the smallest *P*-value). In such cases, a representative variant ranking high in all three analyses was chosen to be listed in the table. Positions correspond to the GRCh37 human genome assembly. Values in parentheses denote the lower and upper bounds of the 95% confidence interval of the effect size estimate. *P*-values for association with ECG morphology were retrieved from `www.ecgenetics.org` [19]. *P*-values with genome-wide significance ($p \leq 5 \times 10^{-8}$ for our GWAS results and $p \leq 1 \times 10^{-10}$ for the results of Verweij *et al* [19] due to multiple testing) are highlighted in bold. *P*-values with suggestive significance in the GWAS ($p \leq 1 \times 10^{-6}$) are highlighted with an asterisk. Chr., Chromosome; Pos., Position; Ref., Reference allele; Alt., Alternative allele; AF, frequency of the alternative allele; assoc., associated; morph., morphology.

Supplementary Table 3: Tissue enrichment analysis results generated by DEPICT using a GWAS P-value threshold of 10^{-6} (showing all entries with nominal P-values $\leq$ 0.05).

MeSH term	Name	MeSH first level term	Nominal P value	False discovery rate
A10.165.450.300	Cicatrix	Tissues	0.02	$\geq$ 0.20
A10.165.450	Granulation Tissue	Tissues	0.02	$\geq$ 0.20
A07.541	Heart	Cardiovascular System	0.03	$\geq$ 0.20
A07.541.560	Heart Ventricles	Cardiovascular System	0.04	$\geq$ 0.20
A07.231.114	Arteries	Cardiovascular System	0.04	$\geq$ 0.20

MeSH, Medical Subject Heading

Supplementary Table 4: Tissue enrichment analysis results generated by DEPICT using a GWAS P-value threshold of 10^{-4} (showing all entries with nominal P-values $\leq$ 0.05).

MeSH term	Name	MeSH first level term	Nominal P value	False discovery rate
A07.541.358	Heart Atria	Cardiovascular System	3.6e-05	$<$0.01
A07.541.358.100	Atrial Appendage	Cardiovascular System	4.5e-05	$<$0.01
A07.541	Heart	Cardiovascular System	7.5e-05	$<$0.01
A07.541.560	Heart Ventricles	Cardiovascular System	2.6e-04	$<$0.01
A10.690	Muscles	Tissues	3.8e-03	$<$0.20
A05.360.319.679.690	Myometrium	Urogenital System	4.9e-03	$<$0.20
A11.329.830	Stromal Cells	Cells	5.8e-03	$<$0.20
A11.329.228	Fibroblasts	Cells	6.1e-03	$<$0.20
A10.690.552.500	Muscle Skeletal	Tissues	6.7e-03	$<$0.20
A10.690.552	Muscle Striated	Tissues	6.7e-03	$<$0.20
A07.231.114	Arteries	Cardiovascular System	7.3e-03	$<$0.20
A02.633.567.850	Quadriceps Muscle	Musculoskeletal System	8.3e-03	$<$0.20
A07.541.510.110	Aortic Valve	Cardiovascular System	8.6e-03	$<$0.20
A07.541.510	Heart Valves	Cardiovascular System	8.6e-03	$<$0.20
A11.329	Connective Tissue Cells	Cells	9.8e-03	$<$0.20
A11.329.629	Osteoblasts	Cells	0.02	$\geq$ 0.20
A10.690.467	Muscle Smooth	Tissues	0.03	$\geq$ 0.20
A06.407.071.140	Adrenal Cortex	Endocrine System	0.03	$\geq$ 0.20
A10.165.450.300.425	Keloid	Tissues	0.04	$\geq$ 0.20
A06.407.071	Adrenal Glands	Endocrine System	0.04	$\geq$ 0.20
A11.872.580	Mesenchymal Stem Cells	Cells	0.04	$\geq$ 0.20
A02.835.583.443	Joint Capsule	Musculoskeletal System	0.05	$\geq$ 0.20
A02.835.583	Joints	Musculoskeletal System	0.05	$\geq$ 0.20
A02.835.583.443.800	Synovial Membrane	Musculoskeletal System	0.05	$\geq$ 0.20
A10.165.450.300	Cicatrix	Tissues	0.05	$\geq$ 0.20
A10.165.450	Granulation Tissue	Tissues	0.05	$\geq$ 0.20

MeSH, Medical Subject Heading

Supplementary Table 5: Results of gProfiler [25] including all loci found in the GWAS with $p \leq 1 \times 10^{-6}$.

Source	Name	ID	P-value
GO:MF	cytoskeletal protein binding	GO:0008092	8.0e-04
	calmodulin binding	GO:0005516	1.8e-03
	[heparan sulfate]-glucosamine N-sulfotransferase activity	GO:0015016	5.2e-03
	actin binding	GO:0003779	6.1e-03
GO:BP	heparin biosynthetic process	GO:0030210	6.9e-04
	heart contraction	GO:0060047	7.8e-04
	heart process	GO:0003015	9.9e-04
	cardiac muscle contraction	GO:0060048	1.1e-03
	muscle system process	GO:0003012	1.3e-03
	cell communication involved in cardiac conduction	GO:0086065	1.4e-03
	bundle of His cell to Purkinje myocyte communication	GO:0086069	1.4e-03
	heparin metabolic process	GO:0030202	1.7e-03
	cardiac muscle cell action potential	GO:0086001	3.5e-03
	regulation of cardiac muscle contraction	GO:0055117	3.9e-03
	striated muscle contraction	GO:0006941	4.3e-03
	muscle contraction	GO:0006936	6.3e-03
	heparan sulfate proteoglycan biosynthetic process	GO:0015012	8.0e-03
	regulation of striated muscle contraction	GO:0006942	9.7e-03
	cardiac conduction	GO:0061337	1.1e-02
	regulation of heart rate	GO:0002027	1.2e-02
	heparan sulfate proteoglycan metabolic process	GO:0030201	1.5e-02
	regulation of muscle system process	GO:0090257	1.6e-02
	regulation of cardiac muscle cell contraction	GO:0086004	1.7e-02
	regulation of actin filament-based movement	GO:1903115	2.8e-02
	regulation of heart rate by cardiac conduction	GO:0086091	2.8e-02
	regulation of membrane depolarization	GO:0003254	3.2e-02
	action potential	GO:0001508	4.5e-02
GO:CC	sarcomere	GO:0030017	1.4e-03
	myofibril	GO:0030016	2.3e-03
	contractile fiber	GO:0043292	2.8e-03
	calcium- and calmodulin-dependent protein kinase complex	GO:0005954	3.3e-03
	Z disc	GO:0030018	4.6e-03
	I band	GO:0031674	6.7e-03
	voltage-gated sodium channel complex	GO:0001518	4.5e-02
KEGG	Melanogenesis	KEGG:04916	1.7e-02
	Glycosaminoglycan biosynthesis - heparan sulfate / heparin	KEGG:00534	2.6e-02
REAC	Phase 0 - rapid depolarisation	REAC:R-HSA-5576892	5.5e-05
	Muscle contraction	REAC:R-HSA-397014	3.8e-03
	Interaction between L1 and Ankyrins	REAC:R-HSA-445095	5.0e-03
	Cardiac conduction	REAC:R-HSA-5576891	1.4e-02
	CaMK IV-mediated phosphorylation of CREB	REAC:R-HSA-111932	3.4e-02
HP	Ventricular tachycardia	HP:0004756	1.3e-02
	Abnormality of synovial bursa morphology	HP:0025231	4.6e-02
	Bursitis	HP:0025232	4.6e-02

GO, Gene Ontology; MF, Molecular Function; BP, Biological Process; CC, Cellular Component; REAC, Reactome Pathway Database; HP, Human Phenotype Ontology

Supplementary Table 6: Results of gProfiler [25] including all loci with $p \leq 1 \times 10^{-4}$.

Source	Name	ID	P-value
GO:MF	cytoskeletal protein binding	GO:0008092	6.4e-05
	transmembrane transporter binding	GO:0044325	5.9e-04
	actin binding	GO:0003779	1.1e-03
	actin filament binding	GO:0051015	1.5e-02
	heparan sulfate sulfotransferase activity	GO:0034483	2.0e-02
GO:CC	sarcoplasm	GO:0016528	2.7e-04
	sarcomere	GO:0030017	2.8e-04
	sarcoplasmic reticulum membrane	GO:0033017	3.0e-04
	Z disc	GO:0030018	3.6e-04
	sarcolemma	GO:0042383	5.7e-04
	myofibril	GO:0030016	8.7e-04
	I band	GO:0031674	9.4e-04
	cytoskeleton	GO:0005856	1.3e-03
	sarcoplasmic reticulum	GO:0016529	1.3e-03
	contractile fiber	GO:0043292	1.4e-03
	cytoplasm	GO:0005737	1.9e-03
	cell junction	GO:0030054	1.6e-02
	supramolecular fiber	GO:0099512	4.2e-02
	supramolecular polymer	GO:0099081	4.8e-02
	calcium ion-transporting ATPase complex	GO:0090534	5.0e-02
KEGG	Adrenergic signaling in cardiomyocytes	KEGG:04261	3.9e-06
	Dilated cardiomyopathy	KEGG:05414	1.0e-03
	Oxytocin signaling pathway	KEGG:04921	8.8e-03
	cAMP signaling pathway	KEGG:04024	9.5e-03
	Arrhythmogenic right ventricular cardiomyopathy	KEGG:05412	1.3e-02
	Cardiac muscle contraction	KEGG:04260	2.8e-02
	Hypertrophic cardiomyopathy	KEGG:05410	3.4e-02
	Amphetamine addiction	KEGG:05031	5.0e-02
REAC	Cardiac conduction	REAC:R-HSA-5576891	4.2e-02
WP	Calcium regulation in cardiac cells	WP:WP536	4.2e-02
HP	Heart block	HP:0012722	1.6e-04
	Ventricular tachycardia	HP:0004756	1.7e-04
	Supraventricular arrhythmia	HP:0005115	8.5e-04
	Cardiac conduction abnormality	HP:0031546	2.3e-03
	Ventricular arrhythmia	HP:0004308	2.6e-03
	Sudden death	HP:0001699	2.8e-03
	Sudden cardiac death	HP:0001645	5.7e-03
	Autosomal dominant inheritance	HP:0000006	1.2e-02
	Dilated cardiomyopathy	HP:0001644	1.3e-02
	Cardiac arrest	HP:0001695	1.6e-02
	Atrioventricular block	HP:0001678	1.8e-02
	Sick sinus syndrome	HP:0011704	1.9e-02
	Reduced systolic function	HP:0006673	2.0e-02
	Abnormal atrioventricular conduction	HP:0005150	3.1e-02

GO:BP and CORUM were skipped for space reasons. GO, Gene Ontology; MF, Molecular Function; BP, Biological Process; CC, Cellular Component; KEGG, Kyoto Encyclopedia of Genes and Genomes; REAC, Reactome Pathway Database; WP, WikiPathways; HP, Human Phenotype Ontology

Supplementary Table 7: Association of DOSI and excess DOSI with delta age.

Metric	Univariate		Multivariate (age, sex, assessment centre)		Multivariate (full)	
	Effect size	*P*-value	Effect size	*P*-value	Effect size	*P*-value
DOSI	-0.52 (-0.65, -0.38)	1.7e-13	0.07 (-0.06, 0.21)	0.28	0.20 (0.07, 0.34)	3.6e-03
eDOSI	0.34 (0.20, 0.49)	2.9e-06	0.09 (-0.04, 0.23)	0.17	0.22 (0.09, 0.36)	1.4e-03

Values in the parentheses denote the lower and upper bounds of the 95% confidence intervals of the effect size estimate.

Supplementary Table 8: Loci associated with "abnormal" ECGs (as automatically labelled by the ECG machine).

Chr.	Gene	rsID	Pos.	Ref.	Alt.	AF	Adj.	LOR	*P*-value
3	*RYBP*	rs9883587	72,383,495	G	A	0.76	basic	0.09 (0.06, 0.13)	4.5e-07*
							full	0.09 (0.06, 0.13)	1.1e-06
	ROBO1	rs115880984	78,675,814	G	A	0.02	basic	-0.28 (-0.40, -0.17)	7.8e-07*
							full	-0.25 (-0.37, -0.14)	2.0e-05

Two GWAS were run with abnormal vs. normal ECGs as binary phenotype. One adjusted for age, sex, genotyping array, and UKB assessment centre, whereas the other one included more covariates for extended adjustment (see Methods for a full list of covariates). Positions correspond to the GRCh37 human genome assembly. Values in parentheses denote the lower and upper bounds of the 95% confidence interval of the effect size estimate. *P*-values with suggestive significance are highlighted with an asterisk. Chr., Chromosome; Pos., Position; Ref., Reference allele; Alt., Alternative allele; AF, frequency of the alternative allele; Adj., Adjustment; LOR, Log odds ratio;

Supplementary Data

Supplementary Data 1 DEPICT geneset enrichment analysis with a P-value threshold of 1×10^{-6}.

Supplementary Data 2 DEPICT geneset enrichment analysis with a P-value threshold of 1×10^{-4}.

Supplementary Data 3 Smallest P-values of variants found by our analysis (GWAS with regular adjustment) in the vicinity (within 1 Mb) of loci associated with parental longevity [26] and leukocyte telomere lengths [27].

Supplementary Data 4 Results of mediation analyses for BMI, MAP, and diagnosed hypertension as individual mediators were determined with the Pingouin Python package [18] and with 5,000 bootstrap iterations per test.

References

1. Attia, Z. I. *et al.* Age and sex estimation using artificial intelligence from standard 12-lead ECGs. *Circulation: Arrhythmia and Electrophysiology* **12,** e007284 (2019).
2. Ioffe, S. & Szegedy, C. *Batch normalization: Accelerating deep network training by reducing internal covariate shift* in *International conference on machine learning* (2015), 448–456.
3. Kingma, D. P. & Ba, J. Adam: A method for stochastic optimization. *arXiv preprint arXiv:1412.6980* (2014).
4. Sarrazin, S., Lamanna, W. C. & Esko, J. D. Heparan sulfate proteoglycans. *Cold Spring Harbor perspectives in biology* **3,** a004952 (2011).
5. Poulain, F. E. & Yost, H. J. Heparan sulfate proteoglycans: a sugar code for vertebrate development? *Development* **142,** 3456–3467 (2015).
6. Zhang, R. *et al.* Heparan sulfate biosynthesis enzyme, Ext1, contributes to outflow tract development of mouse heart via modulation of FGF signaling. *PLoS One* **10,** e0136518 (2015).
7. Vite, A., Li, J. & Radice, G. L. New functions for alpha-catenins in health and disease: from cancer to heart regeneration. *Cell and tissue research* **360,** 773–783 (2015).
8. Frey, N. *et al.* Calsarcin-2 deficiency increases exercise capacity in mice through calcineurin/NFAT activation. *The Journal of clinical investigation* **118,** 3598–3608 (2008).
9. Martin, R. I. *et al.* Genetic variants associated with risk of atrial fibrillation regulate expression of PITX2, CAV1, MYOZ1, C9orf3 and FANCC. *Journal of molecular and cellular cardiology* **85,** 207–214 (2015).
10. Shah, S. *et al.* Genome-wide association and Mendelian randomisation analysis provide insights into the pathogenesis of heart failure. *Nature communications* **11,** 1–12 (2020).
11. Clausen, A. G., Vad, O. B., Andersen, J. H. & Olesen, M. S. Loss-of-Function Variants in the SYNPO2L Gene Are Associated With Atrial Fibrillation. *Frontiers in cardiovascular medicine* **8** (2021).
12. Ellinor, P. T. *et al.* Meta-analysis identifies six new susceptibility loci for atrial fibrillation. *Nature genetics* **44,** 670–675 (2012).
13. Li, A. *et al.* Silencing of the Drosophila ortholog of SOX5 in heart leads to cardiac dysfunction as detected by optical coherence tomography. *Human molecular genetics* **22,** 3798–3806 (2013).
14. Liu, Y. *et al.* High expression levels and localization of Sox5 in dilated cardiomyopathy. *Molecular Medicine Reports* **22,** 948–956 (2020).
15. Washkowitz, A. J., Gavrilov, S., Begum, S. & Papaioannou, V. E. Diverse functional networks of Tbx3 in development and disease. *Wiley Interdisciplinary Reviews: Systems Biology and Medicine* **4,** 273–283 (2012).
16. Van Eif, V. W. *et al.* Genome-wide analysis identifies an essential human TBX3 pacemaker enhancer. *Circulation research* **127,** 1522–1535 (2020).
17. Yang, P. *et al.* βII spectrin (SPTBN1): biological function and clinical potential in cancer and other diseases. *International journal of biological sciences* **17,** 32 (2021).
18. Vallat, R. Pingouin: statistics in Python. *J. Open Source Softw.* **3,** 1026 (2018).
19. Verweij, N. *et al.* The genetic makeup of the electrocardiogram. *Cell systems* **11,** 229–238 (2020).
20. DAgostino Sr, R. B. *et al.* General cardiovascular risk profile for use in primary care: the Framingham Heart Study. *Circulation* **117,** 743–753 (2008).
21. National Health Service. *What's your heart age?* `https://www.nhs.uk/conditions/nhs-health-check/check-your-heart-age-tool/` (2022).
22. Hippisley-Cox, J., Coupland, C., Robson, J. & Brindle, P. Derivation, validation, and evaluation of a new QRISK model to estimate lifetime risk of cardiovascular disease: cohort study using QResearch database. *Bmj* **341** (2010).

23. Pyrkov, T. V. *et al.* Longitudinal analysis of blood markers reveals progressive loss of resilience and predicts human lifespan limit. *Nature communications* **12,** 1–10 (2021).

24. Wang, M., Gao, J., Shi, Y. & Zhao, X. A genome-wide association and polygenic risk score study on abnormal electrocardiogram in a Chinese population. *Scientific reports* **11,** 1–11 (2021).

25. Raudvere, U. *et al.* g: Profiler: a web server for functional enrichment analysis and conversions of gene lists (2019 update). *Nucleic acids research* **47,** W191–W198 (2019).

26. Pilling, L. C. *et al.* Human longevity: 25 genetic loci associated in 389,166 UK biobank participants. *Aging (Albany NY)* **9,** 2504 (2017).

27. Codd, V. *et al.* Polygenic basis and biomedical consequences of telomere length variation. *Nature genetics* **53,** 1425–1433 (2021).
